# Gamified Cognitive Bias Modification Interventions for Psychiatric Disorders: Review

**DOI:** 10.2196/11640

**Published:** 2018-10-25

**Authors:** Melvyn Zhang, Jiangbo Ying, Guo Song, Daniel SS Fung, Helen Smith

**Affiliations:** 1 National Addiction Management Service Institute of Mental Health Singapore Singapore; 2 Department of Developmental Psychiatry Institute of Mental Health Singapore Singapore; 3 Family Medicine and Primary Care Lee Kong Chian School of Medicine Nanyang Technological University Singapore Singapore Singapore

**Keywords:** attention bias, cognitive bias, gamification, psychiatry

## Abstract

**Background:**

Automatic biases, such as attentional biases and avoidance and interpretative biases, have been purported to be responsible for several psychiatric disorders. Gamification has been considered for cognitive bias modification, mainly to address the core issues of diminishing motivation to train over time, as bias modification intervention tasks tend to be highly repetitive. While a prior review has suggested how gamification strategies could be applied to such tasks, there remains a lack of systematic evaluation of gamified cognitive bias modification interventions in the literature.

**Objective:**

The objective of this review is to understand the overall effectiveness of a gamified approach for cognitive bias modification and inform future research that seeks to integrate gamification technologies into existing conventional bias modification interventions.

**Methods:**

To identify the relevant articles for our review, the following search terminologies were used: (“cognitive bias” OR “attention bias” OR “interpret* bias” OR “approach bias” OR “avoidance bias”) AND (“training” OR “modification” OR “practice” OR “therapy”) AND (“gamification” OR “game elements” OR “game” OR “gaming” OR “game mechanics”). PubMed, MEDLINE, PsycINFO, and Scopus databases were searched systematically for articles published after 2000. Articles were included if they described a gamified cognitive bias modification task and included participants with underlying psychopathological symptoms. Data were systematically extracted from the identified articles, and a qualitative synthesis was performed.

**Results:**

Four studies evaluated gamified cognitive bias modification interventions. Two studies included participants with anxiety symptoms, one with affective symptoms, and one with alcohol problems. The conventional visual probe task paradigm was used in 3 studies, and the attentional visual search task paradigm was used in the last study. We found gaming elements incorporated to include that of animations, sounds, feedback, and a point-scoring system for response time and difficulty. Of the 4 identified studies, only 2 reported their gamified interventions to be effective.

**Conclusions:**

Our review is the first to systematically synthesize the evidence for gamified cognitive bias modification interventions. The results arising from our review should be considered in the future design and conceptualization of gamified cognitive bias modification interventions.

**International Registered Report Identifier (IRRID):**

RR2-10.2196/10154

## Introduction

Automatic biases are involved in the psychopathologies of several psychiatric disorders, including anxiety and alcohol and tobacco disorders [1-5]. Cognitive biases include attention, approach/avoidance, and interpretative biases, and these biases can be retrained. Modification of these automatic biases could be achieved with tasks such as the visual probe task (which involves the repeated pairing of probes with neutral stimulus) [6], approach/avoidance task (which involves presenting the salient stimulus in a push-away format) [7], or cognitive bias modification for interpretation (which involves training individuals to disambiguate ambiguous scenarios in a positive way) [8]. Prior reviews have synthesized the evidence for cognitive bias modification [9,10]. In a review by Cristea et al [9], 25 trials involving participants with alcohol and tobacco disorder were identified; bias modification was found to be effective for attentional and approach biases, with an effect size of 0.60. Jones et al [10] reviewed meta-analyses and reported that cognitive bias modification was effective for anxiety disorders with effect sizes ranging from 0.13 to 0.74, depressive disorders with effect sizes ranging from 0.35 to 0.85, and appetitive disorders (defined to include eating and addictive disorders) with effect sizes ranging from 0.003 to 0.36.

Most conventional cognitive bias modification interventions have been delivered in the laboratory, but in recent years, rapidly advancing Web technologies have been increasingly adopted. Wiers et al [11] administered a Web-based attention control training and approach bias retraining intervention for 136 participants with problem drinking and reported a reduction in drinking across all the intervention groups. Similarly, William et al [12] harnessed the potential of Web technologies for the delivery of an online cognitive bias modification training and reported it to be effective in reducing depressive and distress symptoms. In addition to Web technologies, mobile technologies are being used to transform the delivery of bias modification interventions. It has been reported that a mobile app could help in improving insomnia symptoms [13].

Just as technology has transformed the mechanism of delivery of cognitive bias modification intervention, advances in gamification have transformed the nature of conventional cognitive bias modification interventions. Gamification is defined as the use of game-design features in a nongaming context [14], and the term “serious games” refers to games that are designed and built specifically for education, training, or behavioral modification [15]. These technologies have been adopted in health care, and some have been evaluated. Currently, most of these gamified interventions are used in chronic disease rehabilitation and mental health [16], with the most common gamification technique being feedback. Lumsden et al [17] synthesized the evidence for gamification for cognitive assessment and training. The authors reported gamification helped improve engagement in the short and longer term and made the task more attractive. Other studies have found increased self-empowerment [16] and improved existing skills sets [16]. More recently, Lau et al [15] reported in a review that serious games could help improve psychiatric symptoms, with an effect size of 0.55.

Boendermaker et al [18] reviewed how gamification might help address one of the core challenges of conventional interventions, that of motivation to train; these tasks tend to be highly repetitive with a need for multiple training sessions. The article highlighted several potential gamification strategies and explored how they have been used in some studies. The gamification approaches used included the addition of gaming elements to existing tasks, transformation of a conventional task into a serious game, identification of an underlying theory of the intervention and development of a game, addition of a full gaming approach to a conventional task (both intrinsic and extrinsic combinations), and use of over-the-shelf entertainment games. While this review provides timely insight into how gamification strategies have been adapted for bias modification interventions, it was not a systematic review with a database search. Other research on the evaluation of a gamified variant of an attention bias modification task determined that a gamified intervention was effective for anxiety [19]. However, to date, there is no systematic evaluation of gamified cognitive bias modification interventions in the literature. This is needed to understand the overall effectiveness of a gamified approach for cognitive bias modification and inform future research that seeks to integrate gamification technologies into existing conventional bias modification interventions.

The primary aim of our research was to review gamification in cognitive bias modification for psychiatric disorders. Our secondary aim was to identify gamification elements used in cognitive bias modification interventions and evaluate the evidence for their effectiveness by assessing whether the gamified intervention resulted in changes in biases or improvement in secondary outcomes (eg, improvements in anxiety or depressive scores, reduction in the absolute amount of alcohol consumed) and if any motivational improvement was seen.

## Methods

The methods for our systematic review are based on our protocol, published elsewhere [20].

### Search Strategy

To identify relevant articles for the review, the following search terminologies were used: (“cognitive bias” OR “attention bias” OR “interpret* bias” OR “approach bias” OR “avoidance bias”) AND (“training” OR “modification” OR “practice” OR “therapy”) AND (“gamification” OR “game elements” OR “game” OR “gaming” OR “game mechanics”). PubMed, MEDLINE, PsycINFO, and Scopus databases were systematically searched for articles published after 2000; before 2000, there were limited computer-based interventions. When full-text access was not available, the original authors were contacted for their articles.

### Inclusion and Exclusion Criteria

Articles were included if they described a cognitive bias modification intervention in the form of a gamified task and included participants assessed to have underlying psychopathological symptoms such as depressive, anxiety, or addictive symptoms. Articles were excluded if they were opinion pieces, review articles, or design documentation, or described an intervention that used an existing over-the-shelf game. Only English language articles were included.

### Screening, Data Extraction, Sorting, and Selection

All articles identified using the search strategy were downloaded and imported into a reference manager (Endnote X8, Clarivate Analytics). The articles were screened based on their titles and abstracts by two independent authors, MZ and JY. Full copies of the shortlisted articles were evaluated against the inclusion and exclusion criteria with any disagreement resolved by a discussion with the third author (GS).

For relevant articles, the following data were extracted:

- Publication details: authors, study year, and country in which the study was conducted
- Study design (observational or experimental design)
- Sample size
- Type of sample (treatment seeking or community sample)
- Demographics of sample (mean age, gender proportion)
- Psychopathological symptoms of participants and methods of ascertaining psychiatric symptoms
- Details of gamified cognitive bias modification intervention (mechanics of game-play and the conventional cognitive bias modification intervention that the gamified task was based on)
- Primary outcomes and secondary outcomes: effectiveness of gamified cognitive bias modification intervention and any changes in psychiatric symptoms

### Data Integration and Synthesis

A qualitative synthesis of the evidence extracted from the articles was performed. Due to the heterogeneity in the outcomes reported, it was not appropriate to conduct a meta-analytical synthesis.

## Results

### Identified Studies

**Figure 1:**
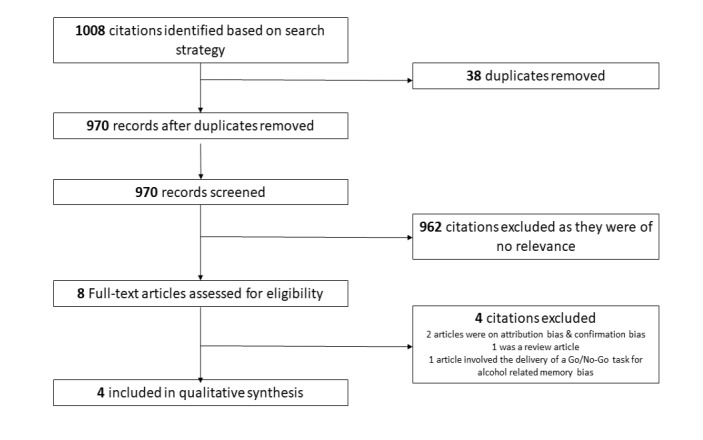
Flowchart of the selection of studies.

The predefined search strategy identified 1008 citations from 4 bibliographic databases; after duplicate articles were excluded, 970 records were screened and, of these, 962 were excluded as not relevant to the topic of interest. Eight full-text articles were downloaded for further evaluation against the inclusion and exclusion criteria. Four citations were excluded as they did not fulfill the inclusion criteria, leaving 4 articles for the qualitative synthesis. Figure 1 provides an overview of the study selection process. For an overview of the characteristics of the selected studies, see Multimedia Appendix 1.

### Characteristics of Identified Studies

Two of the 4 studies identified involved participants with anxiety symptoms [19,21]. One involved participants with alcohol-related problems [4] and one, participants with affective symptoms [22]. All studies were experimental, randomized controlled designs recruiting participants from universities with mean ages of participants 20 to 30 years. All studies were conducted in a western setting. Two studies came from the United States, one from the Netherlands, and another from Belgium. None of the studies used a structured clinical interview to ascertain symptomatology or diagnosis but relied on validated questionnaires (State-Trait Anxiety Inventory [19,21], Mood and Anxiety Symptoms Questionnaire [22], and Alcohol Use Questionnaire and Alcohol Use Disorders Identification Test questionnaire [4]). Three studies based their gamified intervention on the visual probe task and one on the attentional visual search task. Gaming elements integrated into these tasks included animations, sound effects, reward points, time pressure, and levels of complexity.

### Characteristics of a Gamified Cognitive Bias Modification Intervention

Two studies [19,21] used the same app for their intervention, a gamified attention bias modification app based on the conventional dot-probe task. The gamification elements included that of animated characters, a system for points scoring, and sound effects. Two animated characters would appear on the screen simultaneously, both disappearing into a hole. One character would cause a path of grass to rustle behind and participants undertaking the intervention were asked to trace the path in the grass. Based on the author’s description of the gamified intervention, 4 different sounds were played, and different rewards were given depending on participant accuracy and speed. The lowest pitch sound would be played and red jewel awarded for slow response or responses that were least accurate, a medium pitch sound would be played and purple jewel awarded for moderate speed and accuracy, and a high pitch sound would be played and gold jewel awarded if fast and accurate. There was also a feedback sound for incorrect responses. There were 2 variants of the gamified intervention: 25 minutes of training along with 20 minutes of rest or 45 minutes of training with no rest. Points accumulated as the intervention progressed, and feedback was given immediately on completion.

Pieters et al [22] used a gamified app for cognitive bias modification for anxiety symptoms, based on the conventional visual attention task. The game required participants to tap on smiling faces, with smiling faces making up 60% of the faces and disgust faces the remaining 40%. Once the participant tapped on the smiling face, the face bounced up for a short distance and become untappable for 0.5 to 1 second. Participants were instructed to prevent the smiling faces from falling down the screen by tapping on them. Points were awarded to incentivize game play. A point was awarded for tapping the smiling face the first time and 5 points awarded if the same smiling face was tapped a second time. There was negative scoring with the loss of 3 points if a smiling face was not tapped and fell off the screen.

Boendermaker et al [4] used a shot game for attention bias modification, based on the conventional visual probe task. The gamified intervention included a reward system, graphics, animations, sound effects, time pressure, and levels. Their game resembled a slot machine with a coin-based reward system. Like the conventional task, participants were required to identify the probe that replaced the position of the alcohol or neutral image. Participants won bonuses for responding rapidly and were given access to new levels in the game.

Three studies delivered the gamified cognitive bias modification intervention using a mobile device, and Pieters et al [22] used a computer.

### Reasons for Gamification

Two studies described the reasons for inclusion of gamification. The intervention by Boendermaker et al [4] included gaming elements to potentially increase participant motivation to train via the intervention. The authors also sought to determine if the inclusion of gaming elements increased the effectiveness of the conventional visual probe task. Dennis et al [10] were interested in whether the inclusion of gamification changed the effects of the attention bias modification.

### Primary and Secondary Outcomes

Of the 4 studies, 2 [19,21] reported the gamified variant of the cognitive bias modification intervention to be effective. Dennis et al [19] reported that the long-training attention bias modification task resulted in a reduction of threat bias and difficulties individuals had with disengaging from threat-related stimulus. There were also corresponding reductions in the subjective and observed anxiety and stress. Similarly, the authors of the second paper [21] reported that the single session of gamified attention bias modification was effective in improving the performance of the attention bias modification task. However, the authors reported that significant results were observed among females only. Contrary to the findings of Dennis et al and Dennis-Tiwary et al [19,21], Pieters et al [22] reported that their gamified intervention did not result in any reduction in attention biases or associated mood-related measures.

In the study involving participants with alcohol-related problems [22], the gamified variant of the cognitive bias modification task did not reduce attention bias and failed to achieve a decline in overall alcohol consumption. Of importance, the study by Boendermaker et al [4] was the only study that investigated the effects of gamification and motivation of participants in using the training task. Boendermaker et al [2] reported that motivation to train did not increase with the addition of gaming elements. In fact, participants assigned to receiving the gamified variant reported having lower motivation to continue the training task as compared to participants assigned to other conditions.

## Discussion

### Principal Findings

Our review is the first to systematically synthesize the evidence for gamified cognitive bias modification interventions. Of the 4 studies that evaluated a gamified cognitive bias modification intervention, 2 studies included participants with anxiety symptoms, one with affective symptoms, and one with alcohol problems. Gamified interventions were based on the conventional visual probe task in 3 studies and the attentional visual search task in the last study. The gaming elements incorporated into the task included animations, sounds, feedback, and a point-scoring system for response time and difficulty. Two publications discussed their rationales for gamification, one sought to determine if gamification enhances motivation, and one to determine if the gamified attention bias modification was as effective as a conventional modification. Out of the 4 identified studies, 2 studies reported their gamified intervention to be effective. Of significance, these 2 studies used the same app and were from the same research group.

The 4 studies applied gamification across a variety of psychiatric disorders—anxiety and affective and addictive disorders. The conditions that gamified cognitive bias modification interventions target are like those targeted by conventional mobile-based cognitive bias modification interventions. The review by Zhang et al [23] evaluated the published literature and reported that out of 8 identified studies, at least 4 studies used a mobile intervention to target anxiety-related disorders (anxiety and social anxiety disorders). In their review of meta-analyses, Jones et al [10] included 5 meta-analyses that examined anxiety-related outcomes. Thus, anxiety conditions have been extensively investigated in the published literature. This could, therefore, explain why there have been more studies that have applied gamification techniques in increasing the inherent effectiveness of existing conventional training tasks.

All identified studies have based their gamified intervention on the conventional cognitive bias modification intervention, which is of importance, as the conventional cognitive bias modification intervention is the most commonly used task. Three studies were based on the conventional visual probe task and one on the attentional visual search task. In line with the recommended gamification techniques of Boendermaker et al [18], it appears that all 4 studies have used intrinsic integration with evidence-based training task as a basis, given that all 4 studies based their intervention on a conventional task and added gaming elements to that task. Adopting intrinsic integration makes tasks more engaging and might increase the inherent levels of motivation to continue training. Unfortunately, we found no evidence that the adoption of intrinsic integration led to increased motivation to train as only 1 study [4] included motivation to train as an outcome measure, and in that study, there were no improvements.

In keeping with the objectives of the review, we identified some of the common gaming elements that are incorporated in the published gamified interventions: animations, sound effects, point-scoring systems, time pressure, and levels. In their review, Hoffman et al [14] proposed a taxonomy of gamification strategies that could be applied for the evaluation of gamification strategies in apps. The authors used the taxonomy to evaluate stress management apps and found that feedback or performance-orientated strategies were frequently used in the 62 evaluated apps. Like the review of Hoffman et al [14], our findings demonstrated that performance-orientated gamification strategies are used for some of the gamified apps (time pressure and levels in the Boendermaker et al [4] study). Economic gamification strategies are more commonly used, with 4 studies reporting the usage of a point system. The differences in our findings, as compared to that for stress management apps, is not unexpected. Prior research highlighted the importance for designers to carefully consider the gamification techniques used, depending on the nature of the app and how gamification could affect user interaction [14]. Thus, for cognitive bias modification interventions, incorporating feedback might be less feasible, as feedback usually involves a comparison to a set standard or others’ performance. Digital rewards like points might be more tangible, both as an extrinsic motivator and as a surrogate indicator of how well one is performing on the task.

The existing evidence is inconclusive for gamified cognitive bias intervention effectiveness, as only 2 of the 4 studies reported positive findings, but several implications arise from our review. Why gamification is effective in some studies and not others must be determined to guide consideration of which gamification strategies to adopt in an intervention. User perspectives of what makes an app engaging and which strategies result in short and longer term engagement are important to consider in the design of gamified cognitive bias modification interventions. While only 4 studies were identified for this review, Zhang et al [23] found that there were 17 commercial cognitive bias modification apps in the app stores. It might be helpful to analyze the gamification features in commercial cognitive bias modification apps and see if certain gamification features are associated with higher rates of downloads, a surrogate measure of acceptability. Also, a qualitative analysis of the feedback that individuals provide for the gamified commercial apps might be helpful for developers or health care professionals who are creating a new gamified intervention.

### Strengths and Limitations

A major strength of our review is that we systematically identified from the published literature gamified cognitive bias modification interventions and synthesized the evidence for their overall effectiveness. We also identified the gamification strategies that they have adopted. Our review will be of importance for future research seeking to design and evaluate gamified cognitive bias modification interventions, as it provides information about gaming elements that might affect whether interventions are effective. Despite the strengths, there are some limitations. In our review, we were limited to a qualitative synthesis because a meta-analytical synthesis was not appropriate given the heterogeneity in the studies and outcomes reported. Our synthesized results might have limited generalizability, as 2 studies used similar apps and tested the apps in a university sample.

### Conclusions

By identifying gamified cognitive bias modification interventions in the published literature and synthesizing their evidence, our findings have helped bridge the gaps in previous reviews. The results arising from our review should be considered in the future design and conceptualization of gamified cognitive bias modification interventions.

## Appendix

Multimedia Appendix 1 Characteristics of included studies (n=4).
