# Supplementary material for: Gamified Cognitive Bias Modification Interventions for Psychiatric Disorders: Review
Source: JMIR Ment Health. 2018 Oct 25;5(4):e11640. doi: 10.2196/11640 (PMC6229516; doi:10.2196/11640)
Supplement: Multimedia Appendix 1 [file mental_v5i4e11640_app1.pdf]

Multimedia Appendix 1. Characteristics of included studies (n=4).

Study & Country of Study	Study Design & Sample Size	Types of Sample	Demographics of Sample	Psycho-pathological symptoms	Details of Intervention	Primary and Secondary Outcomes
Dennis TA et al. (2014) (1)<br><br>New York, United States of America	Randomised Controlled Study<br><br>Total sample size =78<br><br>Long training condition: 38 participants ; 19 to Attention bias modification training (ABMT) and 19 to placebo training (PT)<br><br>Short training condition: 40 participants; 2 excluded, leaving 18 to ABMT and 20 to PT	Adults recruited from an Introduction to Psychology Course at an urban University	Long training condition: mean age 22.3, 27 females, 11 males<br><br>Short training condition: mean age 20.2, 28 females, 12 males	Anxiety symptoms<br><br>Participants were recruited only if they scored more than 1 SD above the mean for college students on trait anxiety (using the State-Trait Anxiety Inventory).	Gamified mobile application was based on the dot-probe task. Gamification elements included animated characters, points and sound effects.<br><br>In the game-play, two animated characters, one with an angry expression and another with a neutral/mildly positive expression would appear. Thereafter, both would disappear into a hole, with one causing a path of grass to rustle. Participants traced the path of grass.<br><br>Feedback was provided after each trial: red jewel/low pitch sound for slow response, poor accuracy; purple jewel/medium pitch sound for moderate speed & accuracy; gold jewel/high-pitch sound for fast response & accuracy. A high pitch sound was played for errors made. Points were accumulated as the intervention progress.<br><br>The short and long version of the app involved 25 and 45 minutes of training respectively.<br><br>Mechanism of delivery: computer for assessment and iPod Touch (fourth generation) for training	Long-training resulted in reduced threat bias and difficulties with disengaging.<br><br>Both the short and long training resulted in reductions in subjective and observed anxiety and stress.
Boendermaker et al. (2016) (2)<br><br>Amsterdam, The Netherlands	Randomized Controlled Study<br><br>Total sample size=96	Sample of undergraduate students recruited through the university laboratory's website	Mean age 21.2 years, (68/96) 71% were females<br><br>Gamified visual probe task (VPT-G) group: mean age 21.0, 23/33 were females<br><br>Regular visual probe task (VPT-R) group: mean age 21.3, 22/30 were females<br><br>Placebo version of the regular visual	Alcohol Problems<br><br>Participants were recruited if they consumed ≥ 5 standards glasses of alcohol on average per week for males; ≥ 4 for females<br><br>Participants were assessed by means of the Timeline Follow back (TLFB), adapted version of the Alcohol Use Questionnaire (AUQ), and the Alcohol Use Disorders Identification Test (AUDIT)	Gamified mobile application was based on conventional visual probe task. The game elements included reward system, graphics, animations, and sound effects.<br><br>The gamified intervention resembled that of a slot machine. Participants identified the position of the probe that replaced either the substance or neutral stimuli. Participants were rewarded for correct and fast responses (by means of time bonuses and special bonus trials) and new levels.<br><br>Mechanism of delivery: Mobile Device	Decline in attention bias mainly in the regular visual probe training task.<br><br>There was no decline in alcohol consumption after the training.<br><br>Motivation to train decreased in all conditions. This implied that the training task did get boring over time. Participants in the game condition indicated a lower motivation to train as compared to other conditions.

			probe task (VPT-P_ group: mean age 21.4, 23 out of 33 females			
Dennis-Tiwary et al. (2016) (3)<br><br>New York, United States	Randomized Study<br><br>Total sample Size N=42	Adults recruited from an undergraduate research pool at an urban university in New York City, and through Craigslist	Mean age 20.6; 21/42 were females<br><br>ABMT group: 11/19 were females<br><br>PT group: 10/23 females	Anxiety symptoms<br><br>State-Trait Anxiety Inventory was used to screen participants.<br><br>Participants were recruited if they scored +1 standard deviation above the mean for college students on trait anxiety.	Similar app as Dennis TA et al. (2014)	A single session of gamified ABMT improved performance on anxiety-related stress task among females only.
Pieters et al. (2016) (4)<br><br>Ghent, Belgium	Randomized Controlled Study<br><br>Experiment 1: Total Sample= 58<br><br>Assigned to attend positive: 30<br>Assigned to attend negative: 20<br><br>Experiment 2: Total Sample= 86<br><br>Assigned to attend positive: 27<br>Assigned to attend negative: 29<br>Assessment-only group: 26	Undergraduate students	Experiment 1:<br><br>Attend-positive group: mean age 23.9, 23/30 females<br>Attend-negative group:<br>mean age: 23.1, 25/28 females<br><br>Experiment 2:<br><br>Attend-positive group: mean age 21.2, 23/27 females<br>Attend- negative group: mean age:21.2, 23/29 females<br>Assessment-only group: mean age: 23.3, 21/26 females	Affective symptoms<br><br>Assessed by the 30-item version of the Mood and Anxiety Symptoms Questionnaire (MASQ-D30), the Ruminative Response Scale (RRS),	Gamified application based on Intrinsically Motivating Playable Attentional Control Training (IMPACT). Intervention based on conventional visual attention task. Game elements included feedback, time points, difficulty levels and sounds.<br><br>Faces (either smiling or disgust) continuously descend from top to bottom of the screen. Participants were to prevent faces from reaching the bottom of the screen by clicking on the faces twice. Participants in the attend-positive condition were required to clinic on happy faces and ignore disgusted faces; while participants in the attend-negative condition were asked to click on disgusted faces and ignore happy faces. Immediate feedback was provided through time points.<br><br>The task consisted of 15 rounds of 1min each. In the attend-positive condition, 60% of the faces were smiling and 40% were disgusted. This was reversed for the attend-negative condition.<br><br>Mechanism of delivery: computer in laboratory	No significant effect on measures of emotional attentional performance, or on self reported stress, anxiety and depression symptoms

References

1. Dennis TA, O'Toole L. Mental Health on the Go: Effects of a Gamified Attention Bias Modification Mobile Application in Trait Anxious Adults. Clin Psychol Sci. 2014;2(5):576-90.
2. Boendermaker WJ, Sanchez Maceiras S, Boffo M, Wiers RW. Attentional Bias Modification With Serious Game Elements: Evaluating the Shots Game. JMIR Serious Games. 2016;4(2):e20.
3. Dennis-Tiwary TA, Egan LJ, Babkirk S, Denefrio S. For whom the bell tolls: Neurocognitive individual differences in the acute stress-reduction effects of an attention bias modification game for anxiety. Behav Res Ther. 2016;77:105-17.
4. Pieters EK, De Raedt R, Enock PM, De Putter LMS, Braham H, McNally RJ, et al. Examining a Novel Gamified Approach to Attentional Retraining: Effects of Single and Multiple Session Training. Cognitive Therapy and Research. 2016;41(1):89-105.
